# Myeloid-Derived Suppressor Cells (MDSCs) in Haematology

**DOI:** 10.3390/jcm11010187

**Published:** 2021-12-30

**Authors:** Nikoleta Bizymi, Helen A. Papadaki

**Affiliations:** 1 Department of Haematology, University Hospital of Heraklion, 71500 Heraklion, Greece; nikoletabizymi@yahoo.gr; 2 Haemopoiesis Research Laboratory, School of Medicine, University of Crete, 71500 Heraklion, Greece

Myeloid-derived suppressor cells (MDSCs) are immature myeloid cells with immunomodulating properties, mainly acting by suppressing T-cell responses. In humans MDSCs are divided in CD11b^+^CD33^+^HLA-DR^–/low^CD14^+^ cells (monocytic, M-MDSCs); CD11b^+^CD33^+^HLA-DR^–/low^CD15+ or CD66b^+^ cells (granulocytic or polymorphonuclear, G- or PMN-MDSCs) and CD11b^+^CD33^+^HLA-DR^–/low^CD14^−^CD15^−^ cells (early stage MDSCs), while, in mice, they are divided in Gr1^+^CD11b^+^Ly6G^+^Ly6C^low^ cells (PMN-MDSCs) and Gr1^+^CD11b^+^Ly6G^−^Ly6C^hi^ cells (M-MDSCs) [1]. In the current Special Issue of the Journal of Clinical Medicine (JCM) entitled “*Myeloid-Derived Suppressor Cells (MDSCs) in Haematology*”, Vanhaver et al. review the mechanisms of immunosuppression of these cells, as well as the strategies of quantitative and qualitative studying of MDSCs in their paper entitled “*MDSC in Mice and Men: Mechanisms of Immunosuppression in Cancer*”. According to the authors, the main mechanisms include: (a) the depletion of important T cell amino acids (i.e., L-arginine, L-cysteine, L-tryptophan); (b) the induction of oxidative stress via the production of reactive oxygen species (ROS) and nitric oxide (NO); (c) the enhancement of the T regulatory cells (Tregs) expansion and (d) the direct interaction with T cells via the PD1/PDL1 and Fas/FasL pathways [2].

The main body of literature on MDSCs concerns solid tumours, as these cells contribute to oncogenesis, angiogenesis and poor response to therapy [1]. In recent years, however, the implication of MDSCs in haematologic diseases, including immune-mediated and malignant conditions, has been described. Thus, the potential role of these cells as biomarkers and therapeutic targets has started to attract a particular interest in haematology [3]. Indeed, there is already evidence that MDSCs display an altered frequency and/or functionality and can be targeted in haematologic diseases. Thus, this Special Issue aims to cover the current knowledge as it is documented through experimental and clinical studies of MDSCs on this broad spectrum of conditions.

Kapor et al. discuss the interaction of MDSCs with mesenchymal stem cells (MSCs) in myeloid malignancies, in their review entitled “*Myeloid-Derived Suppressor Cells and Mesenchymal Stem/Stromal Cells in Myeloid Malignancies*”. The authors summarised the literature, including acute myeloid leukaemia (AML), myelodysplastic syndromes (MDS), chronic myeloid leukaemia (CML) and Philadelphia chromosome-negative myeloproliferative neoplasms (Ph^−^MPNs). MDSCs are found elevated and immunosuppressive, in in vivo and in vitro studies in the peripheral blood (PB) and bone marrow (BM) of these patients. As MSCs are also thought to have immunomodulating properties and facilitate the development of malignancy in the aforementioned conditions, and as it is already well-known that both cell types act in common ways, the authors suggest that further studies should focus on the MSCs–MDSCs interaction in myeloid malignancies, which can result in the development of new therapeutic approaches for these patients [4].

Moreover, Papafragkos et al. have reviewed the current literature concerning MDSCs in lymphoid malignancies in their paper entitled “*Decoding the Myeloid-Derived Suppressor Cells in Lymphoid Malignancies*”. According to the authors, the literature on the topic is still limited and further research is needed. Special mention is given to the mouse models of lymphomas used for the study of MDSCs. The main studies are presented concerning diffuse large B-cell lymphoma (DLBCL); non-Hodgkin’s lymphoma (NHL); Hodgkin’s lymphoma (HL); B-chronic lymphocytic Leukaemia (B-CLL); follicular lymphoma (FL); marginal zone lymphoma (MZL); mantle cell lymphoma (MCL); monoclonal B-cell lymphocytosis (MBL); small lymphocytic lymphoma (SLL); mucosa-associated lymphoid tissue (MALT); lymphoplasmacytic lymphoma (LPL) and T/natural killer (NK) lymphomas. Overall, MDSCs are found to be elevated in these conditions and related with poor prognosis [5].

Furthermore, in this Special Issue, Wang et al. publish their original research paper entitled “*Elevated M-MDSCs in Circulation Are Indicative of Poor Prognosis in Diffuse Large B-Cell Lymphoma Patients*”. The authors have studied M-MDSCs in newly diagnosed and relapsed DLBCL patients. Not only have they found that M-MDSCs are increased in the PB of the patients, but these cells are also correlated with poor prognosis, as the authors presented a positive correlation of M-MDSCs with the international prognostic index (IPI) score and a negative correlation with the overall survival in their patients. Through further in vitro and in vivo experiments, the authors suggested a possible mechanism of the accumulation of these cells in DLBCL, and this is via interleukin-35 (IL-35), which is known to be increased in this condition [6].

Another interesting field, that of the graft-versus-host disease (GVHD), is covered in the review paper of Demosthenous et al., entitled “*The Role of Myeloid-Derived Suppressor Cells (MDSCs) in Graft-versus-Host Disease (GVHD)*”. The authors present the ways in which MDSCs can have a beneficial therapeutic effect in patients experiencing GVHD, i.e., through the secretion of immunosuppressive cytokines and the induction of Tregs. As a result of their hopeful role in this setting, there are currently studies involving MDSCs in the diagnosis and/or treatment (including the ex vivo administration of MDSCs and in vivo enhancement of their development) of GVHD [7].

The better understanding of the quantitative and functional properties of MDSCs and the mechanisms of their crosstalk with other regulatory (e.g., T cells, NK cells, and MSCs) and malignant cells in haematologic diseases, can facilitate the development of novel therapeutic strategies (i.e., blockage of development, differentiation, depletion and deactivation of MDSCs) and the recognition of novel biomarkers for treatment approaches in the context of precision medicine. In their review paper, entitled “*Resistance to Immune Checkpoint Inhibitors Secondary to Myeloid-Derived Suppressor Cells: A New Therapeutic Targeting of Haematological Malignancies*”, Olivares-Hernández et al. highlight that immune checkpoint inhibitors (ICIs) are promising new agents for haematologic malignancies. The authors suggest that the lack of response in some cases, and the poor outcome, can be in part attributed to the actions of MDSCs. Thus, the measurement in these patients of MDSCs, as well as the targeting of pathways important for these cells, can benefit the patients [8].

In conclusion, as inferred from the points mentioned above, this Special Issue can serve as a great opportunity to highlight the role of MDSCs in the pathophysiology of haematologic diseases, and demonstrate how further research in the field can advance the everyday clinical practice of haematologists, especially in the context of personalised medicine.
